# Effect of Diaphragmatic Breathing Exercise, Jacobson's Relaxation Technique and Dynamic Neuromuscular Stabilization on Gastrointestinal and Psychological Causes of Noncardiac Chest Pain: A Randomized Controlled Trial

**DOI:** 10.1155/prm/8124858

**Published:** 2025-08-14

**Authors:** Anusha Rajesh Suryavanshi, Prem Venkatesan, Ranjan Shetty, Mrudula Pawar

**Affiliations:** ^1^Department of Physiotherapy, Manipal College of Health Professions, Bengaluru, Manipal Academy of Higher Education, Manipal, India; ^2^Cardiology Department, Manipal Hospitals, Old Airport Road, Bengaluru, India

**Keywords:** atypical chest pain, diaphragmatic breathing exercise, DNS, Jacobson's relaxation technique, NCCP

## Abstract

**Objective:** Despite the availability of studies, there is a lack of available literature on the treatment of psychological and gastrointestinal causes of noncardiac chest pain (NCCP). Physiotherapeutic techniques involving diaphragmatic breathing, Jacobson's relaxation and dynamic neuromuscular stabilization (DNS) could address these causes through the activation of parasympathetic system. Hence, this study aims to evaluate the combined effect of diaphragmatic breathing, Jacobson's relaxation and DNS along with pharmacological therapy on patients with gastrointestinal and psychological causes of NCCP.

**Design:** Randomized controlled trial.

**Setting:** Cardiology OPD of a tertiary care hospital.

**Subjects:** Eighty-eight subjects with NCCP.

**Methods:** The intervention group received diaphragmatic breathing, Jacobson's relaxation technique, DNS and pharmacological treatment for 4 weeks. The control group was given pharmacological treatment with patient education. The Beck Depression Inventory (BDI) score, Hamilton Depression Rating Scale (HDRS) score, gastroesophageal reflux disease (GERD) questionnaire score and Numerical Pain Rating Scale (NPRS) score were measured at baseline and after 4 weeks of intervention.

**Results:** The baseline characteristics and outcome measures were assessed, and no significant differences were noted in their mean values (*p* > 0.05). After 4 weeks of intervention, a statistically significant difference was observed in BDI, HDRS, GERD questionnaire score and NPRS in both the groups (*p* < 0.001). However, the GERD questionnaire and NPRS showed statistically significant decrease in the intervention group (GERD questionnaire: 28.39 ± 9.74 and NPRS: 4.57 ± 0.91) compared with the control group (GERD questionnaire: 35.54 ± 12.23 and NPRS: 4.95 ± 0.96) with *p* < 0.05.

**Conclusion:** The diaphragmatic breathing exercises, Jacobson's relaxation technique and DNS are beneficial for reducing GERD symptoms and pain in patients with NCCP. These techniques are safe and cost-effective treatment for addressing the psychological and gastrointestinal causes of NCCP. These can be incorporated along with the pharmacological treatment to enhance the outcomes. However, future researches with long-term follow-ups are required.

**Trial Registration:** Clinical Trials Registry - India: CTRI/2025/02/081294

## 1. Introduction

Noncardiac chest pain (NCCP) is a type of recurrent chest pain that is indistinguishable from ischemic heart pain after the exclusion of cardiac factors [1]. It can have a musculoskeletal, pulmonary, gastrointestinal or psychogenic origin. NCCP has a prevalence of 70% globally [2] and 25% in India [3]. Approximately half of the population presenting to the cardiology department is diagnosed with NCCP [4], of which 60% and 58.7% are gastrointestinal and psychological [5, 6]. Among these, the gastrointestinal cause comprises of majorly gastroesophageal reflux disease (GERD) and minority of patients with oesophageal motility disorders. The psychological cause of NCCP comprises of panic disorder, anxiety and depression [7].

The current pharmacological management for patients with NCCP involves histamine-2 receptor antagonists and proton-pump inhibitors for GERD-related NCCP and muscle relaxants and pain modulators for other non-GERD-related NCCP [8]. NCCP being a heterogenous disorder, a multimodal therapeutic approach is required for its management. At present, there is dearth in literature addressing the gastrointestinal and psychological cause through nonpharmacological therapies. The current treatment options provided for these patients include cognitive behavioural therapy, hypnotherapy, deep friction massage and heat packs, showing varied results [9, 10]. Furthermore, there are limited studies with physiotherapeutic interventions focussing on the gastrointestinal and psychological causes of NCCP.

The existing evidence has described the independent effects of breathing exercises, Jacobson's relaxation and dynamic neuromuscular stabilization (DNS) among older adults with a history of anxiety and depression [11], gastroesophageal reflux pain and chronic pain [12, 13]. A recent systematic review [11] reported that the Jacobson progressive muscle relaxation technique and deep breathing exercises positively affect anxiety, psychological distress and quality of life in hospitalized older adults. In addition, a meta-analysis [12] revealed that, compared with medical management alone, combining breathing exercises with medical management significantly improved symptoms and quality of life in GERD patients.

DNS emphasizes the regulation of breathing patterns, intra-abdominal pressure control, elongation of the spine and centration of joints to activate deep core muscles, including the diaphragm [13]. A randomized controlled trial in patients with GERD [14] reported that DNS leg raise causes changes in upper and lower-oesophageal sphincter pressure, thereby reducing postprandial acid reflux and symptoms of gastroesophageal reflux disorders such as nausea and burning chest pain.

By understanding the efficacy of DNS on GERD outcomes and the effects of breathing exercises and Jacobson's relaxation techniques on psychological outcomes, we hypothesize that a treatment protocol combining these interventions could address the psychological and GERD aspects in NCCP patients. Therefore, this study aimed to evaluate the combined effects of diaphragmatic breathing exercise, Jacobson's relaxation technique and DNS on the psychological and gastrointestinal symptoms of NCCP in comparison with standard care.

## 2. Methods

### 2.1. Study Design

This study is a prospective, randomized, controlled, single-blinded trial that included subjects with NCCP from Cardiology OPD, Manipal Hospital, from May 2023 to October 2023. The protocol was approved by the ethical committee of Manipal Hospital.

#### 2.1.1. Patient Screening Criteria

Among the 210 participants, 88 were recruited from May 2023 to October 2023. Patients were eligible if they presented to the cardiology department with chest pain and were diagnosed with NCCP by a physician or cardiologist after normal ECG during pain and at rest, normal echocardiography and a negative treadmill test [15]. Both male and female participants aged 18–55 years who understood English were included. Patients with acute myocardial infarction, a history of trauma to the thorax, a rise/fall pattern of cardiac troponin values, including myocardial injury, Alzheimer's disease, dementia and a history of spinal surgery were excluded from the study. The included subjects were randomized into two groups via block randomization. The intervention group (*n* = 44) received physiotherapeutic interventions along with pharmacological management, and the control group (*n* = 44) received only medical management with patient education.

### 2.2. Procedure

The RCT followed the CONSORT guidelines. Patients were recruited at the Cardiology Department of Manipal Hospital, Bangalore, after ethical clearance. After screening 210 participants, 88 participants who met all the inclusion and exclusion criteria were randomized to the intervention (exercise) group or the control group. Informed consent was obtained from the participants.

The baseline assessment results of the Beck Depression Inventory (BDI), Hamilton Depression Rating Scale (HDRS), GERD questionnaire and Numerical Pain Rating Scale (NPRS) were recorded for the intervention and control groups. The patients in the intervention group were taught diaphragmatic breathing, Jacobson's relaxation technique and DNS. They were advised to perform the exercises three times a day for 4 weeks along with medical management, as advised by the cardiologist. The patients in the control group received patient education and medical management.

Regular follow-up for exercise adherence was conducted via weekly telephone calls. The number of exercise sessions performed by the patients was recorded. After 4 weeks of intervention, patients were reassessed via the BDI, the HDRS, the GERD questionnaire and the NPRS.

### 2.3. Interventions

The diaphragmatic breathing exercise included breathing slowly through the nose and then out through the mouth via the diaphragm and abdominal muscles. The patient was advised to exercise thrice a day for 4 weeks [16]. Jacobson's relaxation technique involved sequential contraction and relaxation of specific muscle groups to relieve tension in the muscles. The patient was advised to exercise for 10–15 min 3 days per week [17]. During DNS, the patient was advised to lie supine with the hip and knee at the 90–90 position, with the spine upright and relaxed shoulder girdles with alignment of the chest and pelvis. The patient was subsequently asked to perform abdominal breathing for 2 sets of 8 repetitions [13].

### 2.4. Outcome Measures

The demographic variables including age, gender, duration of pain, manual assessment of respiratory motion (MARM), presence of diabetes mellitus, presence of hypertension, ingestion of antacids and consumption of smoking and alcohol were assessed at baseline. The BDI, HDRS, GERD questionnaire and NPRS were administered at baseline and at the end of the fourth week.

MARM was used to examine the breathing pattern of the patients [18]. The patient was in sitting position and the therapist stood behind the patient. The therapist placed his/her hands each on the either sides of the vertebral column over the dorsal thoracic area such that the thumb remained parallel to the spine and fingers spread across. The index and middle finger assessed the thoracic movement and the ring and small finger assessed the abdominal movement during breathing. Imaginary vertical and horizontal lines were drawn to depict thoracic and abdominal movements. The angles of these lines with horizontal axis were measured. The difference between these angles was noted. However, MARM balance was taken as a baseline assessment but was not included as an outcome measure.

BDI was used to measure the presence and severity of depression experienced in the past 2 weeks. It is a 21-item self-report inventory that reflects the cognitive, affective and somatic components of depression used in adolescents and adults. The patient reports each item on a 4-point scale starting from 0 (*absent*) to 3 (*severe*). The total score is 63, and minimal depression, mild depression, moderate depression and severe depression are indicated by the scores from 0 to 13, 14 to 19, 20 to 28 and 29 to 63, respectively. BDI has a good reliability (*r* = 0.93) and internal consistency (α = 91). It also has a high content validity, construct validity, concurrent validity and criterion validity [19].

The HDRS consists of 17 items, each defined by a series of symptoms, and measures both psychic anxiety (mental agitation and psychological distress) and somatic anxiety (physical complaints related to anxiety). Each item is rated on either 5 point or 3 point scale, resulting in a total score of 53. The score of more than 7 signifies depression. It has an interrater reliability of 0.74 and a retest reliability of 0.81. The scale also has a high content, convergent, predictive and discriminant validity [20].

The GERD questionnaire assesses the symptom severity of gastroesophageal reflux disease and measures changes in typical GERD symptoms such as heartburn and regurgitation for the last 1 month. It is a 19-item questionnaire in which the frequency of symptoms is scored from 1 (*no symptoms*) to 5 (*daily symptoms*) and severity from 1 (*not at all*) to 5 (*extremely severe*). It has an interrater reliability of 0.93, internal consistency of α = 0.94 and high validity [21].

The NPRS was used to assess the pain intensity of the patients. It is a subjective measure and the most widely used patient-reported outcome measure for pain. The ordinal 11-point NPRS (0: *no pain* and 10: *most intense pain*) is a valid and most commonly used version with good test–retest reliability (*r* = 0.79–0.96) [22].

### 2.5. Sample Size

At a 5% level of significance (α) with 80% power (1 − β) and an effect size of 0.6 (μ_d_/s_p_), the minimum sample size (per group) required for a two-group comparison involving GERD as the primary outcome across intervention and control groups, is 44 per group.

(1) $$\begin{array}{c} n=\frac{2s_{p}^{2}{\left(Z_{1-\left(\alpha /2\right)}+Z_{1-\beta }\right)}^{2}}{\mu_{d}^{2}}, \end{array}$$

where *s*_*p*_^2^ = ((*s*_1_^2^ + *s*_2_^2^)/2).

Here, *s*_1_^2^ represents the standard deviation in the first group, *s*_2_^2^ represents the standard deviation in the second group and μ_*d*_^2^ is the mean difference between the samples.

### 2.6. Statistical Analysis

The statistical analysis was performed via Jamovi software Version 2.3 [23]. Frequency tables and percentages were used to describe the categorical variables in the study. The quantitative/numerical variables in the study were described using the mean and standard deviation or median and interquartile range (expressed as Quartile 1 and Quartile 3) after an assessment of the skewness. In the intervention group, the paired *t* test was used for within group differences in BDI. However, the Wilcoxon test was used for HDRS, GERD questionnaire and NPRS as there was violation of assumption of normality. Similarly, in the control group, the paired *t* test was used for within-group differences in GERD questionnaire but the Wilcoxon test was used for BDI, HDRS and NPRS. The between-group comparisons were performed using the independent *t* test for BDI, HDRS and GERD questionnaire. However, the Mann–Whitney U test was used for NPRS as the homogeneity of variance was violated.

## 3. Results

**Figure 1:**
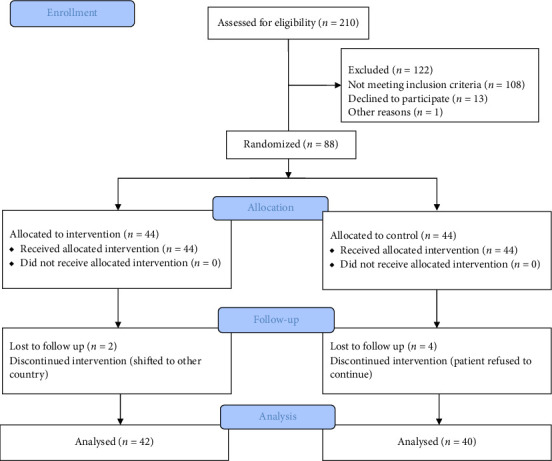
CONSORT flow diagram.

A total of 88 participants who fulfilled the inclusion criteria and agreed to participate in the study were recruited. Forty-four patients were allocated to each group (Figure 1). The mean age of the participants was 42 years, with 37 females and 51 males. The demographics and outcome measures were assessed at baseline, and no significant differences were noted in their mean values (Table 1). The patients were diagnosed with noncardiac chest pain for an average duration of 5 months (control: 4.83 ± 4 and intervention: 4.93 ± 7.4). The demographic variables including gender, presence of diabetes mellitus, presence of hypertension, ingestion of antacids and consumption of smoking and alcohol were compared using the independent *t* test for BDI, HDRS, GERD questionnaire and NPRS (Supporting Information (available here)).

Among the 88 patients, 82 completed the 4-week follow-up, and 6 patients (4 from the control group and 2 from the intervention group) quit the study after consenting to participate. The patients in our study reported 89% adherence to the exercise sessions in the 4 weeks.

The results of the within-group analysis revealed statistically significant changes in the mean values at baseline and after 4 weeks in the intervention group in BDI (17.72 decreased to 13.61), HDRS (19.67 decreased to 12.44), and GERD questionnaire score (47.42 decreased to 28.39) compared with those in the control group BDI (18.84 decreased to 15.48), HDRS (19.64 decreased to 14.08) and GERD questionnaire score (46.73 decreased to 35.54) (Tables 2 and 3). The NPRS score significantly improved in both the control and intervention groups from 6.25 ± 0.670 to 4.95 ± 0.959 and from 6.17 ± 0.621 to 4.57 ± 0.914 (*p* < 0.001), respectively, after 4 weeks of intervention (Tables 2 and 3).

**Figure 2:**
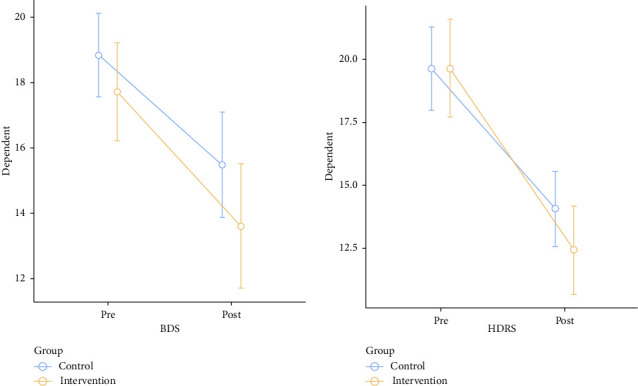
Difference in BDS and HDRS before and after the treatments across the groups.

**Figure 3:**
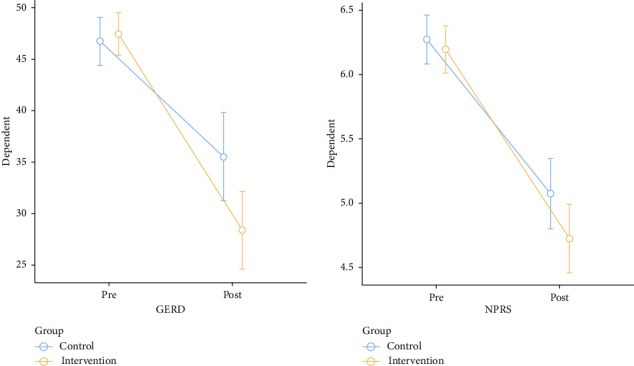
Difference in GERD questionnaire and NPRS before and after the treatments across the groups.

The between-group comparison showed statistically significant improvement in GERD questionnaire and NPRS in the intervention group (Table 4). The graphical representations of the differences in outcomes before and after the treatment in both the groups are presented in Figures 2 and 3.

No adverse effects were reported by any patients in either the intervention or the control group.

## 4. Discussion

The current study aimed to investigate the effects of diaphragmatic breathing, Jacobson's relaxation technique and DNS on the gastrointestinal and psychological causes of NCCP. The primary finding of the study suggests that a significant difference was observed between the intervention group and the control group in the NPRS and GERD questionnaire from baseline to 4 weeks postintervention. In addition, significant improvements were observed within the intervention and control groups in the NPRS, BDI, HDRS and GERD questionnaire scores from baseline to 4 weeks postintervention.

The intervention group demonstrated a significant decrease of 1.60 points in NPRS after 4 weeks, which was above the MCID of 1.5 points, indicating that the treatment is clinically effective for patients with gastrointestinal and psychological causes of NCCP. The current findings are in line with a study reporting a decrease in pain after slow breathing manoeuvres and progressive relaxation techniques [16]. The positive effects on pain reduction in the intervention group may be attributed to reduced pain perception following diaphragmatic breathing exercise and Jacobson's technique, as diaphragmatic breathing plays an important role in pain signalling, autonomic activation, acid base equilibrium and anti-inflammatory processes through activation of the parasympathetic system and an increase in the pain threshold [16, 24, 25]. Relaxation through breathing exercise may help reduce excessive muscle tone, which may restrict blood flow and reduce pain intensity [26].

The intervention group showed a significant change of 19.03 points at 4 weeks and exceeded the MCID of 5 points in the GERD questionnaire, suggesting clinically significant results. Similar findings were reported by Halland et al., who reported favourable outcomes after breathing exercise for 2 days in reducing postprandial reflux [27]. The potential reasons could be the positioning of the diaphragm surrounding the lower-oesophageal sphincter. Another potential reason could be the increased intra-abdominal pressure induced by the leg raising position in DNS. This pressure activation stimulates the diaphragm, which subsequently regulates the lower-oesophageal sphincter and alleviates symptoms in GERD patients [14].

The changes of 4.11 and 7.22 points in the BDI score and HDRS score at 4 weeks exceeded the MCIDs of 3 points and 2 points, respectively, in the intervention group, demonstrating the clinical significance of the treatment in alleviating the psychological causes of the NCCP score. The possible mechanism for this could be vagal activation of the GABA pathway, thereby causing physiological relaxation in the autonomic and neuroendocrine systems with reduced cortisol levels [28]. Similarly, previous studies [29, 30] have shown that the Jacobson progressive relaxation technique and diaphragmatic breathing help lower stress, anxiety, muscle tension, pain and fatigue in older adults and adolescent females with dysmenorrhoea. In addition, these techniques enhance emotion, effectively reduce anxiety and its symptoms and alleviate negative emotions such as depression, stress and anger [31, 32].

The BDI and HDRS scores in the control group patients significantly changed by 3.36 and 5.56 points, respectively, at 4 weeks and exceeded the MCID scores of 3 points and 2 points, respectively. Similarly, there was a significant improvement of 11.19 points in the GERD questionnaire, which exceeded the MCID value of 5 points. Furthermore, the difference was not significant between the groups for psychological outcomes. The positive outcome in control group may be attributed to the physiological benefits of pharmacological management to alleviate symptoms associated with underlying gastrointestinal and psychological causes of NCCP. In addition, patient education was given to the participants in the control group, which could have a potential beneficial effect on the psychological aspects among them. The reassurance about the patient condition during the session could result in an enhanced behaviour and attitude towards the chest pain. This would further improve self-efficacy among these patients.

Compared with independent pharmacological interventions, diaphragmatic breathing, Jacobson's relaxation technique and DNS combined with pharmacological treatment significantly ameliorated pain and GERD parameters in NCCP patients. However, a nonsignificant difference was observed in their psychological parameters. The physiological changes that occur with physiotherapeutic interventions could be attributed to the differences in improvements between the groups.

Along with the physiological impact of the treatment, the patient perception and other sociodemographic factors such as age, gender and economic status influence the symptoms related to NCCP. A previous study concluded that the patients lacked proper understanding about their illness and struggled to perceive the mechanism behind noncardiac causes of chest pain [33]. This resulted in restricted activity among them, further affecting their work and relationships negatively. These socioeconomic factors also affect the treatment allocation, which varies in most of the hospitals. The current study was carried out in a tertiary care hospital, with an easy access for the cardiac investigations, in order to rule out the cardiac causes of NCCP. These patients with NCCP underwent further assessments, and focussed interventions were planned for them. The information provided to the patients through patient education in both the groups lead to a better understanding of the cause of the disease, thus improving the psychological health. Furthermore, the physiotherapy treatment for the patients in the experimental group resulted in an early return to their previous health status.

This is the first study with the specific interventions for gastrointestinal and psychological causes of NCCP. The follow-up through telephone calls improves the feasibility, adherence and cost-effectiveness of the treatment strategies. Future multicentred trials could guide in forming healthcare policies to integrate physiotherapy treatment for the management of patients with NCCP.

This study has several limitations. First, it was performed in one geographical location; thus, it cannot be generalized to an entire population. Second, the level of severity of psychological involvement was not subdivided within the groups. Finally, the study lacked long-term follow-up.

## 5. Conclusion

The diaphragmatic breathing exercises, Jacobson's relaxation technique and DNS, along with pharmacological treatment, aid in alleviating gastroesophageal symptoms in patients with NCCP.

Future studies with a longer follow-up duration and progression of exercise in the intervention group could be performed.

## Figures and Tables

**Table 1:** Baseline characteristics.

Baseline demographics and outcome measures	Intervention group (*n* = 44) Mean ± SD	Control group (*n* = 44) Mean ± SD	*p* value
Age (years)	41.4 ± 11	42.36 ± 11	0.64
Duration of pain (months)	4.82 ± 4	4.93 ± 7.4	0.84
MARM balance	49.75 ± 5.3	50.3 ± 6.5	0.70
BDI	17.72 ± 2.74	18.84 ± 3.42	0.25
HDRS	19.67 ± 4.87	19.64 ± 3.41	0.33
GERD questionnaire	47.42 ± 6.17	46.73 ± 5.72	0.52
NPRS	6.17 ± 0.62	6.25 ± 0.67	0.41

*Note: n*, number of participants.

Abbreviations: BDI, Beck Depression Inventory; GERD, gastroesophageal reflux disease; HDRS, Hamilton Depression Rating Scale; MARM, Manual Assessment of Respiratory Motion; NPRS, Numerical Pain Rating Scale; SD, standard deviation.

**Table 2:** Effect of treatment on the intervention group.

Outcome	Pretreatment (*n* = 44)		Posttreatment (*n* = 42)		*p* value
	Mean ± SD	Median (*Q*_1_, *Q*_3_)	Mean ± SD	Median (*Q*_1_, *Q*_3_)	
BDI	17.72 ± 2.74	18.00 (16.00, 20.00)	13.61 ± 3.72	14.00 (11.00, 16.75)	< 0.001^a∗^
HDRS	19.67 ± 4.87	20.00 (17.00, 21.00)	12.44 ± 3.60	13.00 (9.25, 15.00)	< 0.001^b∗^
GERD questionnaire	47.42 ± 6.17	47.00 (43.25, 51.75)	28.39 ± 9.74	26.00 (23.00, 30.00)	< 0.001^b∗^
NPRS	6.17 ± 0.62	6.00 (6.00, 7.00)	4.57 ± 0.91	5.00 (4.00, 5.00)	< 0.001^b∗^

*Note: n*, number of participants; Q1, first quartile; Q3, third quartile.

Abbreviations: BDI, Beck Depression Inventory; GERD, gastroesophageal reflux disease; HDRS, Hamilton Depression Rating Scale; NPRS, Numerical Pain Rating Scale; SD, standard deviation.

^a^Student's *t*.

^b^Wilcoxon *w*.

^∗^Significant value, if *p* < 0.05.

**Table 3:** Effect of treatment on the control group.

Outcome	Pretreatment (*n* = 44)		Posttreatment (*n* = 40)		*p* value
	Mean ± SD	Median (*Q*_1_, *Q*_3_)	Mean ± SD	Median (*Q*_1_, *Q*_3_)	
BDI	18.84 ± 3.42	20.00 (17.00, 22.00)	15.48 ± 4.18	17.00 (12.00, 19.00)	< 0.001^b∗^
HDRS	19.64 ± 3.41	20.00 (18.00, 22.00)	14.08 ± 3.77	14.00 (11.00, 17.00)	< 0.001^b∗^
GERD questionnaire	46.73 ± 5.72	46.00 (43.50, 50.50)	35.54 ± 12.23	37.50 (26.00, 44.75)	< 0.001^a∗^
NPRS	6.25 ± 0.67	6.00 (6.00, 7.00)	4.95 ± 0.96	5.00 (4.00, 6.00)	< 0.001^b∗^

*Note: n*, number of participants; Q1, first quartile; Q3, third quartile.

Abbreviations: BDI, Beck Depression Inventory; GERD, gastroesophageal reflux disease; HDRS, Hamilton Depression Rating Scale; NPRS, Numerical Pain Rating Scale; SD, standard deviation.

^a^Student's *t*.

^b^Wilcoxon *w*.

^∗^Significant value, if *p* < 0.05.

**Table 4:** Between group differences after 4 weeks of treatment.

Outcomes	Intervention group (*n* = 42) Mean ± SD	Control group (*n* = 40) Mean ± SD	*p* value
BDI	13.61 ± 3.72	15.48 ± 4.18	0.14^a^
HDRS	12.44 ± 3.60	14.08 ± 3.77	0.16^a^
GERD questionnaire	28.39 ± 9.74	35.54 ± 12.23	0.01^a∗^
NPRS	4.57 ± 0.91	4.95 ± 0.96	0.04^b∗^

*Note: n*, number of participants.

Abbreviations: BDI, Beck Depression Inventory; GERD, gastroesophageal reflux disease; HDRS, Hamilton Depression Rating Scale; NPRS, Numerical Pain Rating Scale; SD, standard deviation.

^a^Student's *t*.

^b^Mann–Whitney U.

^∗^Significant value, if *p* < 0.05.

## Data Availability

The data that support the findings of this study are available from the corresponding author upon reasonable request.

## Supporting Information

The comparison of demographics for BDI, HDRS, GERD questionnaire and NPRS is provided in the supporting information.
