# Supplementary material for: Effect of Diaphragmatic Breathing Exercise, Jacobson's Relaxation Technique and Dynamic Neuromuscular Stabilization on Gastrointestinal and Psychological Causes of Noncardiac Chest Pain: A Randomized Controlled Trial
Source: Pain Res Manag. 2025 Aug 14;2025:8124858. doi: 10.1155/prm/8124858 (PMC12373466; doi:10.1155/prm/8124858)
Supplement: Supporting Information — Additional supporting information can be found online in the Supporting Information section. [file 8124858.f1.docx]

**SUPPLEMENTARY MATERIAL**

**Comparison of demographics for BDI**

|  |  | Pre BDI (n=88) | |  | Post BDI (n=82) | |  |
| --- | --- | --- | --- | --- | --- | --- | --- |
|  |  | Mean±SD | Median (Q_1_,Q_3_) | p-value | Mean±SD | Median (Q_1_,Q_3_) | p-value |
| Gender | F | 20.26±8.17 | 18 (17.50, 21.00) | 0.28^b^ | 14.81±4.31 | 16 (11.00, 18.00) | 0.86^a^ |
|  | M | 18.28±3.34 | 18 (16.00, 20.00) |  | 14.59±3.91 | 14 (11.00, 17.75) |  |
| Diabetes Mellitus | No | 18.00±3.14 | 18 (16.00, 20.50) | 0.14^b^ | 14.94±4.06 | 16 (11.00, 18.00) | 0.48^a^ |
|  | Yes | 22.54±10.22 | 20 (18.00, 22.00) |  | 13.90±4.17 | 14.50 (10.25, 17.50) |  |
| Hyper-tension | No | 18.82±2.90 | 18 (17.00, 21.00) | 0.50^b^ | 15.58±3.75 | 17 (12.00, 18.50) | 0.04^b^ |
|  | Yes | 20.79±10.41 | 18.50 (16.25, 21.50) |  | 12.73±4.15 | 12 (9.50, 14.50) |  |
| Antacid ingestion | No | 18.88±7.99 | 18 (15.25, 20.00) | 0.68^a^ | 13.96±3.71 | 14 (11.00, 17.00) | 0.18^a^ |
|  | Yes | 19.64±2.94 | 20 (18.00, 22.00) |  | 15.63±4.39 | 17 (12.00, 19.00) |  |
| Smoking/ Alcohol history | No | 18.04±3.60 | 18 (15.00, 21.00) | 0.17^a^ | 14.61±4.14 | 15 (11.50, 18.00) | 0.88^a^ |
|  | Yes | 20.52±7.97 | 19 (17.00, 21.00) |  | 14.80±4.07 | 15.50 (10.75, 18.25) |  |

^a, student’s t ; b, welch’s t; c,mann-whitney u^

^n,number of participants; SD,standard deviation; Q1,first quartile; Q3,third quartile^

^BDI,Beck Depression Inventory^

**Comparison of demographics for HDRS**

|  |  | Pre HDRS (n=88) | |  | Post HDRS (n=82) | |  |
| --- | --- | --- | --- | --- | --- | --- | --- |
|  |  | Mean±SD | Median (Q_1_,Q_3_) | p-value | Mean±SD | Median (Q_1_,Q_3_) | p-value |
| Gender | F | 20.27±4.23 | 20 (18.00, 22.00) | 0.45^a^ | 13.71±4.14 | 14 (10.00, 16.00) | 0.59^a^ |
|  | M | 19.40±3.63 | 20 (17.00, 21.00) |  | 13.09±3.41 | 13 (10.25, 15.75) |  |
| Diabetes Mellitus | No | 19.49±3.81 | 20 (17.50, 21.00) | 0.34^a^ | 13.45±4.05 | 14 (10.00, 16.00) | 0.85^a^ |
|  | Yes | 20.75±4.18 | 20.50 (17.75, 22.25) |  | 13.20±2.66 | 13.50 (12.25, 14.75) |  |
| Hyper-tension | No | 20.15±4.01 | 20 (18.00, 21.00) | 0.41^a^ | 13.81±3.78 | 15 (11.00, 16.50) | 0.38^a^ |
|  | Yes | 19.08±3.80 | 18 (16.00, 22.00) |  | 12.64±3.61 | 12 (9.50, 14.50) |  |
| Antacid ingestion | No | 18.52±3.81 | 18 (16.00, 20.00) | 0.01^a^ | 12.67±3.93 | 12.50 (9.00, 15.25) | 0.15^a^ |
|  | Yes | 21.27±3.55 | 21 (20.00, 22.75) |  | 14.32±3.38 | 15 (13.00, 16.50) |  |
| Smoking/ Alcohol history | No | 19.68±4.23 | 18 (17.00, 21.00) | 0.81^a^ | 13.17±3.42 | 13 (10.50, 15.00) | 0.68^a^ |
|  | Yes | 19.95±3.59 | 20 (19.00, 21.75) |  | 13.65±4.17 | 14.50 (10.00, 17.00) |  |

^a, student’s t ; b, welch’s t; c,mann-whitney u^

^n,number of participants; SD,standard deviation; Q1,first quartile; Q3,third quartile^

^HDRS,Hamilton Depression Rating Scale^

**Comparison of demographics for GERD**

|  |  | Pre GERD (n=88) | |  | Post GERD (n=82) | |  |
| --- | --- | --- | --- | --- | --- | --- | --- |
|  |  | Mean±SD | Median (Q_1_,Q_3_) | p-value | Mean±SD | Median (Q_1_,Q_3_) | p-value |
| Gender | F | 46.48±6.35 | 46 (43.50, 51.00) | 0.62 ^a^ | 33.77±12.16 | 32.50 (25.25, 44.75) | 0.25 ^a^ |
|  | M | 47.26±5.67 | 46.50 (43.25, 50.75) |  | 30.22±10.85 | 26 (24.00, 36.00) |  |
| Diabetes Mellitus | No | 46.00±6.20 | 45 (43.00, 50.50) | 0.06^a^ | 30.63±11.07 | 27 (24.00, 35.25) | 0.37^a^ |
|  | Yes | 49.11±4.62 | 50 (45.50, 51.50) |  | 33.47±12.11 | 36 (22.50, 40.50) |  |
| Hyper-tension | No | 47.00±6.32 | 46 ( 43.00, 51.00) | 0.98^a^ | 30.34±10.76 | 27.50 (24.25, 35.75) | 0.17^a^ |
|  | Yes | 46.95±5.27 | 47.50 (43.75, 51.00) |  | 34.65±11.96 | 35.00 (23.75, 42.25) |  |
| Antacid ingestion | No | 46.46±4.99 | 46 (43.00, 50.75) | 0.33^a^ | 32.35±11.24 | 29.50 (24.00, 40.25) | 0.26^a^ |
|  | Yes | 49.27±8.92 | 48 (45.50, 55.50) |  | 28.00±11.87 | 25 (21.50, 29.00) |  |
| Smoking/ Alcohol history | No | 48.28±4.52 | 47 (45.00, 51.00) | 0.15^a^ | 34.21±12.28 | 30.50 (25.00, 45.50) | 0.14^a^ |
|  | Yes | 46.06±6.59 | 44.50 (42.75, 50.00) |  | 29.71±10.53 | 26 (23.50, 36.00) |  |

^a, student’s t ; b, welch’s t; c,mann-whitney u^

^n,number of participants; SD,standard deviation; Q1,first quartile; Q3,third quartile^

^GERD,Gastroesophageal Reflux Disease questionnaire^

**Comparison of demographics for NPRS**

|  |  | Pre NPRS (n=88) | |  | Post NPRS (n=82) | |  |
| --- | --- | --- | --- | --- | --- | --- | --- |
|  |  | Mean±SD | Median (Q_1_,Q_3_) | p-value | Mean±SD | Median (Q_1_,Q_3_) | p-value |
| Gender | F | 6.32±0.75 | 6 (6.00, 7.00) | 0.20^c^ | 4.78±0.89 | 5 (4.00, 5.00) | 0.81^c^ |
|  | M | 6.16±0.55 | 6 (6.00, 6.00) |  | 4.74±0.99 | 5 (4.00, 5.00) |  |
| Diabetes Mellitus | No | 6.26±0.66 | 6 (6.00, 7.00) | 0.49^c^ | 4.76±0.98 | 5 (4.00, 5.00) | 0.91^c^ |
|  | Yes | 6.15±0.61 | 6 (6.00, 6.75) |  | 4.75±0.89 | 5 (4.00, 5.00) |  |
| Hyper-tension | No | 6.24±0.66 | 6 (6.00, 7.00) | 0.93^c^ | 4.85±0.93 | 5 (4.00, 5.00) | 0.32^c^ |
|  | Yes | 6.25±0.58 | 6 (6.00, 7.00) |  | 4.62±0.94 | 5 (4.00, 5.00) |  |
| Antacid ingestion | No | 6.23±0.59 | 6 (6.00, 7.00) | 0.89^c^ | 4.74±1.00 | 5 (4.00, 5.00) | 0.85^c^ |
|  | Yes | 6.22±0.75 | 6 (6.00, 7.00) |  | 4.79±0.833 | 5 (4.00, 5.00) |  |
| Smoking/ Alcohol history | No | 6.25±0.71 | 6 (6.00, 7.00) | 0.81^c^ | 4.68±1.0. | 5 (4.00, 5.00) | 0.48^c^ |
|  | Yes | 6.21±0.59 | 6 (6.00, 7.00) |  | 4.82±1.00 | 5 (4.00, 5.00) |  |

^a, student’s t ; b, welch’s t; c,mann-whitney u^

^n,number of participants; SD,standard deviation; Q1,first quartile; Q3,third quartile^

^NPRS,Numerical Pain Rating Scale^
